# Stability Comparison of Implants with Alumina Sandblasting and Acid Etching Surface Treatment: A Retrospective Cohort Study

**DOI:** 10.3390/jcm14030740

**Published:** 2025-01-23

**Authors:** Song-I Back, Myung-Jin Chung, Ho-Gul Jeong, Ji-Hyun Min

**Affiliations:** 1 Department of Dental Hygiene, College of Health and Medical Sciences, Cheongju University, 298 Daesung-ro, Cheongwon-gu, Cheongju 28503, Chungcheongbuk-do, Republic of Korea; tkgk00qw@naver.com (S.-I.B.); rari98@cju.ac.kr (H.-G.J.); 2 Guardians Dental Clinic, 11 Gyesung 3-gil, Dangjin 31770, Chungcheongnam-do, Republic of Korea; cmjiny@naver.com

**Keywords:** dental implants, implant stability quotient, insertion torque value

## Abstract

**Objectives:** This retrospective cohort study aimed to compare and evaluate the 1-year stability of two Korean implant brands, Osstem and Toplan, both treated with alumina- sandblasting and acid- etching (SA) surface modification. **Methods:** This retrospective analysis evaluated patients with American Society of Anesthesiologists grade I or II, >20 years, with alveolar bone volume suitable for implant placement, who received immediate or delayed placement after extraction, and with Osstem (*n* = 57) or Toplan (*n* = 87) implants. The insertion torque value (ITV) measured on the day of implant placement and the implant stability quotient (ISQ) measured on the day of implant placement, 1 month post-surgery, and 2–3 months after implantation were analyzed. **Results:** Both implants had significantly increased ISQs over time, and the ISQs did not significantly differ between Osstem and Toplan implants at any time point. Osstem implants showed significantly higher ISQs in D2 than in D3 bone, and in the mandible than in the maxilla at all time points. Toplan implants with diameters >4.0 mm showed higher initial ISQs. Osstem implants showed a significant correlation between ITV and ISQ on the day of placement (r = 0.349, *p* < 0.01) but not at later time points. For Toplan implants, no significant correlation was confirmed between ITV and ISQ at any time point. At the 1-year follow-up, both implants were still providing functional service. **Conclusions:** Osstem and Toplan implants with SA surface treatment showed a high level of stability for 1 year, and no significant difference in stability was observed between the two implants. Both implants are considered clinically reliable products.

## 1. Introduction

Dental implants are widely used to restore the function and aesthetics of lost teeth [1]. Implant success is determined by several factors. An implant’s shape, including its length and diameter, the bone quality, and the surgical method employed can significantly impact implant success [2,3,4]. Therefore, to avoid implant failure, various parameters such as implant shape, patient bone quality, and surgical method should be considered. In addition, efforts are being made to enhance the surface area and the hydrophilic surface treatments to improve the stability of implants [5,6].

Surface treatment of an implant by roughening the surface of the implant using alumina and then forming stable irregularities through acid etching (SA) promotes bone fusion [6]. Implants with rough surfaces have larger surface areas than those with other processed surfaces, showing higher bone fusion and faster bone healing [7,8].

One method to evaluate the primary stability of an implant is to measure the insertion torque value (ITV; N/cm), which is an effective parameter of the degree of stability during implantation [9]. However, evaluating the overall bone fusion process of an implant is inaccurate, and whether there is a threshold level of ITV that can predict a successful implant is unclear [10].

One representative method of evaluating implant stability is the implant stability quotient (ISQ). The ISQ is an index used to evaluate the stiffness and deformation of an implant and a bone complex and measures the stability of an implant through resonance frequency analysis, and a higher ISQ score indicates higher stability. The ISQ device is easy to use in clinical practice and has high reliability; therefore, it is widely used to evaluate the stability of an implant [4,11].

This study aimed to evaluate the stability of two implant systems treated with an SA surface. The aim was to compare implant stability quotients measured on the day of implant placement, 1 month post-surgery, and 2–3 months post-surgery, respectively, and to assess whether the implants still provided functional services at 1-year follow-up. The hypothesis was that there would be no significant differences in the stability between the two systems.

## 2. Materials and Methods

### 2.1. Study Design and Patient Data Collection

We retrospectively reviewed the medical data of patients who visited the G Dental Clinic in Dangjin, Chungcheongnam-do, between January 2022 and July 2023. The minimum sample size for implants was calculated as 128 using G*Power (version 3.1.9.7, Heinrich Heine Universität Düsseldorf, Düsseldorf, Germany) with an effect size of 0.25, α = 0.05, and power = 0.80. Data collection was conducted in December 2023 and January 2024.

The inclusion criteria were healthy patients aged >20 years, with American Society of Anesthesiologists grade I or II, with occlusal relationship with normal occlusal in one tooth, with alveolar bone volume suitable for implant placement, and who underwent delayed (at least 2 months) implant placement after extraction or immediate implant placement after extraction. Those who were smoking more than 10 cigarettes per day [12], with systemic disease that may affect bone metabolism (osteoporosis), with periodontal disease with confirmed overall swelling and bleeding in the gingiva, who were pregnant, who were using an immunosuppressant, who were undergoing head and neck radiotherapy, with uncontrolled diabetes, with contraindications for simple oral surgery, and who did not meet the follow-up baseline criteria were excluded. Only cases performed by a single dentist with >15 years of clinical experience were included in this study. Based on these criteria, 97 patients (average age, 53.74 ± 11.87 years) were selected. The age range of participants was 28–80 years. Finally, 57 fixtures in group 1 (Osstem TS III; Osstem Implant Co., Ltd., Seoul, Republic of Korea) and 87 fixtures in group 2 (Toplan T01; Toplan Co., Ltd., Seoul, Republic of Korea) in the oral cavity were selected for the analysis. The type of implant was chosen by the patient based on preference, and the implant size was chosen by the dentist after diagnosis.

Implants were classified according to the sex of the study participants, alveolar bone quality, implant placement location, implant placement timing, diameter, and length. Bone quality was classified as D1 to D4, according to Misch’s classification method [13]. Implant lengths ranged from 7.0 to 11.5 mm in group 1 and from 7.0 to 12.0 mm in group 2, and implant diameters ranged from 4.0 to 5.0 mm in group 1 and from 3.6 to 5.0 mm in group 2 (Table 1).

### 2.2. Implant Placement: Surgical Protocol

The surgical plan was established using cone beam computed tomography before the implant procedure. The length of the implant was conditioned by the height of the base of the bone, and the diameter of the implant was determined according to the width of the alveolar process. All surgeries were performed in the operating room using a completely aseptic protocol with infection control. The patient gargled with 10 mL of an oral cleaner containing 0.2% chlorhexidine for 1 min before surgery, and extracorporeal disinfection was performed using cotton balls with povidone iodine and chlorhexidine.

The surgical site was anesthetized with lidocaine hydrochloride with epinephrine injection (1:100,000; Huons Co., Ltd., Seongnam-si, Republic of Korea) and articaine with epinephrine injection (1:100,000; Huons Co., Ltd., Seongnam-si, Republic of Korea), after which the mucosa-periosteal bone flap was elevated. If a lesion was observed around the tooth, it was removed using a surgical curette. The implant insertion process was performed according to the manufacturer’s instructions. The implant was placed 1 mm deeper than the bone level (1 mm subcrestal), and the healing abutment was installed after implantation. The patients were given the following medications after implant placement surgery: amoxicillin hydrate 500 mg and loxoprofen sodium hydrate 68.1 mg tid for 7 days, methylprednisolone 4 mg tid for 6 days, and esomeprazole magnesium trihydrate 22.25 mg od for 7 days. Prosthetic restorations were installed only if ISQ ≥60 was met 2–3 months after implant surgery.

### 2.3. Measurement of ITV

ITVs were measured using a torque wrench calibrated to newtons per centimeter at the time of implant placement. All implant insertion procedures were performed according to the manufacturer’s instructions. The ITVs were recorded as soon as the final location within the bone was reached, and we investigated which sections the ITV of the patient belonged to, as follows: <30, 30–40, 40–50, or >50 N/cm. The torque wrench is a mechanical tool, so a certain level of measurement error may occur when the operator operates the torque wrench by hand, therefore it was interpreted as a range.

### 2.4. Measurement of Implant Stability Quotient

Resonance frequency analysis measurements of implant stability were performed using the ISQ device Osstell™ (Osstell AB, Stampgatan, Göteborg, Sweden), according to the manufacturer’s instructions. Implant stability was expressed as ISQ. The ISQs were recorded in triplicate using Smartpeg™ (Osstell AB) fixed to the implant at a manufacturer-recommended torque of 4–6 N/cm. The primary, secondary, and tertiary measurements were performed according to the implant examination schedule of this dental hospital: on the day of implant placement (ISQ t1), one month after implantation (ISQ t2), and two to three months after implantation (ISQ t3). Measurements were obtained twice in the mesiodistal and buccolingual directions of the implant, and the average value was recorded. Prosthetic restorations were installed when ISQ ≥60 was met, according to the criteria of the ISQ device manufacturer and values suggested in previous studies [14].

### 2.5. Implant Survival and Failure

Implant survival was defined as an implant that remained in place at the 1-year follow-up appointment and supported the restoration. Panoramic radiography was performed at the 1-year follow-up examination. Implant failure was defined as the removal of dental implants at the 1-year follow-up appointment owing to loss of bone fusion, mobility, persistent pain, fractures, and/or extensive bone loss [12].

### 2.6. Statistical Analyses

Normal distribution of the ISQs was confirmed using the Kolmogorov–Smirnov test. To determine the difference in ISQs between group 1 and 2 implants, an independent-samples *t*-test was performed. Differences in ISQs according to the implantation method, implant diameter, implant length, and bone mass were confirmed using the Kruskal–Wallis and Bonferroni correction post hoc test or the Mann–Whitney test. Differences according to the number of ISQ measurements were confirmed using Friedman’s analysis of variance and Wilcoxon’s signed-rank post hoc tests. The correlation between ITV and ISQs was analyzed using Spearman’s rank correlation. All data analyses were performed with two-sided tests using SPSS version 29.0 (IBM Corp., Armonk, NY, USA), and *p* < 0.05 was considered statistically significant.

## 3. Results

### 3.1. Classification by ITV

The distribution of ITV (insertion torque value) between the two groups is compared, showing that the majority of implants fall within the 30–40 N/cm range. (Table 2).

### 3.2. Comparison of Mean ISQ Between Group 1 and 2 Implants

**Figure 1:**
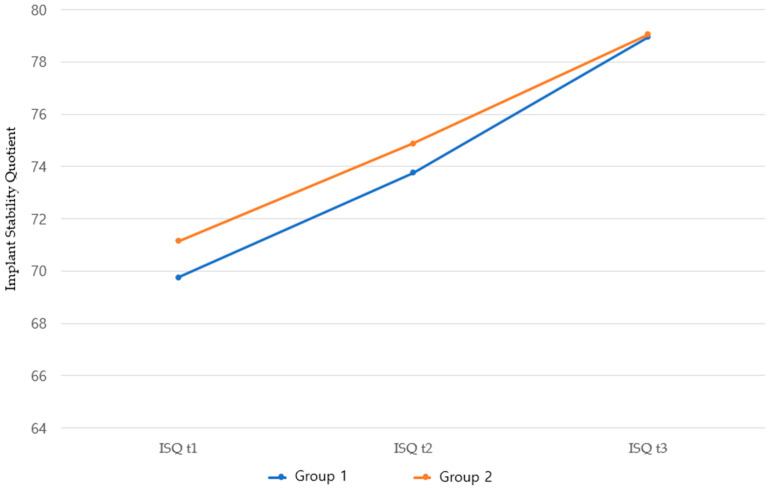
Comparison of ISQ over time between groups 1 and 2. ISQ t_1_, ISQ t_2_, and ISQ t_3_ are the implant stability quotients measured on the day of implant placement, 1 month post-surgery, and 2–3 months post-surgery, respectively.

The comparison of the mean ISQs between group 1 and 2 implants at three time points (implant placement date, 1 month post-surgery, and 2–3 months post-surgery) showed no statistically significant difference at any time point. Both implants showed significant increases in the ISQs over time (Table 3, Figure 1).

### 3.3. Changes in ISQs According to Measurement Timing and Factors

Group 1 and 2 implants differed among ISQ t_1_, ISQ t_2_, and ISQ t_3_ in all factors, except for the group 1 implant with a diameter ≤4.0 mm (*p* = 0.002 or *p* < 0.001). Group 1 implants differed significantly in terms of bone quality and implant location between the ISQ t_1_ and ISQ t_2_ groups (*p* = 0.001 and *p* < 0.001, respectively). Group 2 implants differed significantly in ISQ t_1_ depending on the diameter (*p* = 0.021) (Table 4).

### 3.4. Correlations Between ITV and ISQ

Significant correlations were found between the ITV and ISQ t_1_ in group 1 implants (r = 0.349, *p* < 0.01) but no correlation was found between ITV and ISQ t_1_ in group 2 implants (r = 0.026, *p* > 0.05). In addition, for group 1 implants, significant correlations were found between ISQ t_1_ and ISQ t_2_ and between ISQ t_2_ and ISQ t_3_ (*p* < 0.01) (Table 5). In group 2 implants, significant correlations were found between ISQ t_1_ and ISQ t_2_ (*p* < 0.001), between ISQ t_1_ and ISQ t_3_ (*p* < 0.001), and between ISQ t_2_ and ISQ t_3_ (*p* < 0.001) (Table 6).

### 3.5. Implant Survival and Failure

Panoramic radiographs were performed at the 1-year follow-up examination for both implants. No dental implants were removed at the 1-year follow-up examination due to loss of bone fusion, mobility, persistent pain, fractures, and/or extensive bone loss.

## 4. Discussion

SA surface treatment is an implant surface treatment technology that promotes fusion of the implant and alveolar bone [6]. In previous studies, implants with rough surfaces exhibited high bone fusion and rapid bone healing [7,8]. In this study, we compared and evaluated the stability of two Korean implants that underwent SA surface treatment in order to help consumers make an informed choice.

The primary stability of an implant is the immediate stability obtained upon implantation. It refers to the degree to which an implant is mechanically fixed to the bone and can be measured mainly using ITV and ISQ [9,11]. The initial stability of an implant is important because a higher initial stability leads to better bone adhesion and a higher long-term success rate [11]. Therefore, to increase the initial stability of the implant, different variables such as the implant shape, patient bone quality, and surgical method should be considered. In this study, the ITV of group 1 implants showed a correlation with the ISQ during implantation but there was no significant relationship with the ISQ thereafter. However, the ITV of the group 2 implants did not correlate with the ISQ value at any time point. The ITV has been recognized as a valid parameter for determining implant stability during implantation [11]. As the insertion torque increases, the presence and volume of high-density cortical bone play a significant role in enhancing the initial stability of the implant [15]. This is because cortical bone offers higher mechanical strength and resistance, allowing the implant to achieve a more secure and stable fixation during placement. Additionally, tactile information obtained from surgical twist drills can assist in selecting the initial insertion torque to achieve implant stability [9]. However, it has been suggested that it is not possible to confirm whether there is a correlation between ITV and ISQ or whether they are independent and incomparable methods [16]. Combining the results of the previous study with those of this study, the assumption that a higher ITV always indicates higher primary stability is not entirely accurate, as bone quality and quantity can vary significantly between patients. Additionally, in this study, the variability in bone quality and quantity for both group 1 and group 2 implants suggests that surgical technique may have inherent limitations. In this study, a significant increase in ISQs over time was observed in both the group 1 and 2 implants. According to the literature review, ISQ is influenced by various factors, including measurement direction, gender, implant location, implant diameter and length, implant design, surgical technique, insertion torque, cortical bone thickness, and bone quality and type [17]. The healing period after surgery (20–60 days) is a critical phase due to bone remodeling around the implant. In D3/D4 bone density, there is a relatively higher risk of micromovement [11]. The group 1 implant had significantly higher ISQ t1 and ISQ t2 values in the D2 bone than in the D3 bone and significantly higher ISQs in the mandible than in the maxilla at all time points, suggesting that differences in bone quality and density have an effect on initial implant stability. According to previous studies, the primary ISQ value in D2 bone was higher than in D3 bone, indicating that higher bone density results in higher primary ISQ values compared to lower bone density [18]. For group 2 implants, the group with a diameter >4.0 mm had a higher initial ISQ than the group with a diameter ≤4.0 mm. This is consistent with a previous study showing that larger diameters increase the contact area between the bone and the implant, thereby improving primary stability [19].

The secondary stability is the stability at which implants and bones are biologically combined over time and are mainly formed during osseointegration. Osseointegration occurs as bone cells grow on the surface of the implant and generally proceeds for 1 to 3 months post-surgery [20]. It has been suggested that an ISQ ≥60–65 and an ITV ≥20–45 N/cm are appropriate conditions for loading a single implant crown [14]. In addition, the ISQ values observed in both implant systems indicate reliable primary stability at implantation. Moreover, these values suggest the potential for favorable outcomes in immediate loading after tooth extraction [21]. Although group 2 implants showed slightly higher mean ISQ values compared to group 1, the difference was not statistically significant. These findings align with previous studies [22] that reported similar ISQ trends across comparable implant designs. Clinically, the consistent increase in ISQ values over time indicates successful osseointegration, supporting the suitability of both implants for early loading protocols.

Small differences in ISQs may be caused by minor differences in implant design or surface texture; however, SA surface treatment was consistent in both systems, which is likely a major factor contributing to the osseointegration and initial stability. Among the recent advancements in implant surface treatment technologies, SA surface treatment has gained widespread recognition for its ability to enhance surface roughness, thereby promoting osseointegration and facilitating the adhesion and proliferation of osteoblasts. This technique is cost-effective, provides reliable outcomes, and is widely utilized globally. However, potential drawbacks, such as residual alumina particles that may affect biocompatibility or increase the risk of bacterial adhesion, should be carefully considered [23].

A previous retrospective analysis reported a 10-year cumulative survival rate of 94.8% for SA-treated implants [24]. Early complications, such as infection and initial osseointegration failure, significantly influenced survival rates, and marginal bone loss exceeding 1 mm within the first year was strongly associated with these complications [24]. However, in this study, the two systems of group 1 and group 2 are equivalent in terms of primary stability, are suitable for clinical use, and are likely to contribute to patient satisfaction. In addition, neither implant showed radiolucency around the fixture at the 1-year follow-up, and there were no cases of mobility or pain. One year after implantation, the implants were still providing functional services.

This study has limitations. It was difficult to sufficiently control for confounding variables because this study analyzed retrospective data, and there were limitations in ensuring the accuracy and completeness of the study data. Additionally, future studies with larger sample sizes are needed to allow for more detailed analyses based on various factors such as specific implant locations and implant placement methods. Furthermore, there is a limitation in that comparative studies between different implant surface treatment methods should be included. Therefore, in the future, a prospective study with more samples and additional methods is needed to evaluate the long-term clinical success rate of various types of implants.

## 5. Conclusions

Both group 1 and 2 implants subjected to SA surface treatment showed a high level of primary stability when measured using ISQs. The primary stability did not significantly differ between the two implants, which is believed to provide credibility to the early loading and clinical use of both implants.

## Figures and Tables

**Table 1:** Results of comparing the characteristics of the two implants used in this study.

Characteristics	Group 1 (Osstem Implant)	Group 2 (Toplan Implant)
Manufacturer	Osstem Implant Co., Ltd.	Toplan Co., Ltd.
Model name	Osstem TS III	Toplan T01
Body shape	Conical, 1.5° taper	Conical, Taper–-straight–-taper
Thread shape	Triangular thread	Triangular thread
Pitch height (mm)	0.8 (double)	0.9 (double)
Thread height (mm)	0.45	0.4
Implant–abutment interface	Internal hexagon	Internal hexagon
Inclination angle of the thread flank (°)	40	30
Surface treatment	SA	SA
Microthreads	None	None
Figure of the implant	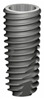	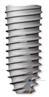

SA, sandblasting with alumina and acid etching. It was written based on the following: diameter, 4.0; length, 10 mm.

**Table 2:** Classification of group 1 and 2 implants by ITV.

Classification of ITVs	Group 1 (*n* = 57)	Group 2 (*n* = 87)
<30 N/cm	3 (5.26)	3 (3.45)
30–40 N/cm	38 (66.67)	65 (74.71)
40–50 N/cm	14 (24.56)	15 (17.24)
>50 N/cm	2 (3.51)	4 (4.60)

Values are presented as *n* (%). ITV, insertion torque value.

**Table 3:** Comparison of mean ISQ of group 1 and 2 implants.

**Groups**	ISQ t_1_	ISQ t_2_	ISQ t_3_	*p*-Value ^†^
Group 1 (*n* = 57)	69.76 ± 12.30	73.74 ± 10.10	78.94 ± 9.12	<0.001
Group 2 (*n* = 87)	71.13 ± 7.86	74.88 ± 7.35	79.03 ± 5.64	<0.001
*p*-value ^‡^	0.416	0.462	0.944	

Values are presented as mean ± standard deviation. ^†^ *p*-values obtained from repeated-measures analysis of variance. ^‡^ *p*-values obtained from independent -samples *t*-test. ISQ t_1_, ISQ t_2_, and ISQ t_3_ are the implant stability quotients measured on the day of implant placement, 1 month post-surgery, and 2–3 months post-surgery, respectively.

**Table 4:** Comparison of ISQs by factors and timing of measurement.

		*n*	Group 1			*p*-Value ^†^	*n*	Group 2			*p*-Value ^†^
			ISQ t_1_	ISQ t_2_	ISQ t_3_			ISQ t_1_	ISQ t_2_	ISQ t_3_	
Sex	Man	33	71.00 ^a^ (64.25, 5.00)	76.00 ^b^ (69.00, 0.50)	82.00 ^c^ (77.75, 5.50)	**<0.001**	71	72.50 ^a^ (69.00, 6.50)	74.50 ^b^ (70.00, 0.50)	78.00 ^c^ (75.00, 2.50)	**<0.001**
	Woman	24	72.50 ^a^ (65.50, 79.38)	73.25 ^b^ (67.50, 2.00)	76.50 ^b^ (71.63, 5.63)	**0.002**	16	72.50 ^a^ (63.63, 9.50)	75.50 ^b^ (71.25, 0.00)	80.75 ^c^ (76.75, 5.00)	**<0.001**
	*p*-value ^‡^		0.437	0.903	0.132			0.641	0.507	0.167	
Bone type	D2	30	73.50 ^a^ (70.38, 80.13)	78.50 ^b^ (71.50, 83.38)	82.00 ^c^ (77.88, 86.00)	**<0.001**	42	74.25 ^a^ (68.75, 9.50)	77.00 ^b^ (71.38, 80.50)	80.00 ^c^ (77.50, 2.13)	**<0.001**
	D3	27	67.00 ^a^ (56.50, 74.00)	69.00 ^a^ (66.00, 78.00)	77.50 ^b^ (71.00, 85.00)	**<0.001**	45	72.00 ^a^ (66.25, 5.00)	72.50 ^b^ (70.00, 80.00)	77.00 ^c^ (74.25, 5.75)	**<0.001**
	*p*-value ^‡^		**0.001**	**0.001**	0.095			0.159	0.156	0.465	
Implant location	Maxilla	24	64.75 ^a^ (56.13, 74.00)	69.00 ^a^ (65.25, 75.75)	76.50 ^b^ (71.00, 84.13)	**<0.001**	45	72.00 ^a^ (68.00, 5.00)	74.00 ^b^ (70.25, 80.00)	78.00 ^c^ (74.75, 5.75)	**<0.001**
	Mandible	33	73.00 ^a^ (70.00, 79.75)	78.50 ^b^ (71.50, 82.75)	82.00 ^c^ (78.75, 86.00)	**<0.001**	42	74.25 ^a^ (67.00, 9.50)	75.50 ^b^ (70.75, 80.50)	79.75 ^c^ (75.38, 81.63)	**<0.001**
	*p*-value ^‡^		**0.001**	**<0.001**	**0.027**			0.219	0.743	0.643	
Implant placement timing	Immediately implanted	27	71.00 ^a^ (67.00, 75.00)	76.00 ^b^ (66.50, 81.00)	80.00 ^c^ (73.50, 82.50)	**<0.001**	47	72.50 ^a^ (68.50, 6.50)	74.50 ^b^ (71.00, 0.00)	79.00 ^c^ (75.00, 82.00)	**<0.001**
	Delayed placement	30	71.00 ^a^ (63.13, 79.00)	73.50 ^b^ (68.88, 80.63)	82.50 ^c^ (77.50, 86.25)	**<0.001**	40	72.00 ^a^ (65.63, 6.50)	75.00 ^b^ (70.00, 2.38)	80.25 ^c^ (75.00, 84.75)	**<0.001**
	*p*-value ^‡^		0.725	0.949	0.088			0.871	0.821	0.855	
Implant diameter	≤4.0 mm	3	71.00 ^a^ (60.00, 73.00)	76.00 ^a^ (43.00, 78.50)	77.00 ^a^ (42.50, 80.00)	0.717	27	70.00 ^a^ (62.00, 4.00)	74.00 ^b^ (69.50, 0.50)	79.50 ^c^ (75.00, 85.50)	**<0.001**
	>4.0 mm	54	71.00 ^a^ (64.88, 77.50)	74.50 ^b^ (68.88, 81.00)	81.25 ^c^ (74.00, 86.00)	**<0.001**	60	74.50 ^a^ (69.13, 7.25)	75.00 ^b^ (71.13, 0.00)	78.75 ^c^ (75.00, 82.38)	**<0.001**
	*p*-value ^‡^		0.579	0.532	0.138			**0.021**	0.666	0.797	
Implant length	≤10 mm	17	70.00 ^a^ (63.75, 77.50)	72.00 ^b^ (69.00, 80.50)	81.00 ^b^ (73.75, 85.50)	**<0.001**	32	72.00 ^a^ (64.25, 75.00)	72.50 ^b^ (70.00, 79.13)	77.75 ^c^ (75.00, 81.38)	**<0.001**
	>10 mm	40	71.25 ^a^ (65.38, 77.38)	76.00 ^b^ (68.50, 80.88)	80.50 ^c^ (74.00, 85.75)	**<0.001**	55	72.50 ^a^ (68.50, 77.50)	75.50 ^b^ (71.00, 80.50)	79.50 ^c^ (75.00, 85.00)	**<0.001**
	*p*-value ^‡^		0.663	0.972	0.727			0.197	0.137	0.420	

Values are presented as median (25%, 75%). ^†^ *p*-value obtained by Friedman’s analysis of variance. ^‡^ *p*-value obtained by Mann–Whitney test. ^a,b,c^ Different letters indicate significant differences in Wilcoxon’s signed-rank post hoc analysis. ISQ t_1_, ISQ t_2_, and ISQ t_3_ are the implant stability quotients measured on the day of implant placement, 1 month post-surgery, and 2–3 months post-surgery, respectively. The bold indicates statistical significance.

**Table 5:** Correlation between ITV and ISQ in group 1.

	ITV	ISQ t_1_	ISQ t_2_	ISQ t_3_
ITV	1			
ISQ t_1_	0.349 **	1		
ISQ t_2_	0.204	0.666 ***	1	
ISQ t_3_	−0.190	0.202	0.340 **	1

** *p* < 0.01, *** *p* < 0.001. Spearman’s rank correlation analysis was used. ISQ t_1_, ISQ t_2_, and ISQ t_3_ are the implant stability quotients measured on the day of implant placement, 1 month post-surgery, and 2–3 months post-surgery, respectively.

**Table 6:** Correlation between ITV and ISQs in group 2.

	ITV	ISQ t_1_	ISQ t_2_	ISQ t_3_
ITV	1			
ISQ t_1_	0.026	1		
ISQ t_2_	−0.011	0.557 ***	1	
ISQ t_3_	0.075	0.359 ***	0.781 ***	1

*** *p* < 0.001. Spearman’s rank correlation analysis was used. ISQ t_1_, ISQ t_2_, and ISQ t_3_ are the implant stability quotients measured on the day of implant placement, 1 month post-surgery, and 2–3 months post-surgery, respectively.

## Data Availability

The data presented in this study are available upon request from the corresponding author.
